# Leriche syndrome diagnosed due to polytrauma: a case report

**DOI:** 10.1186/s12245-022-00411-x

**Published:** 2022-02-04

**Authors:** Genki Yoshimura, Ryo Kamidani, Tomotaka Miura, Hideaki Oiwa, Yosuke Mizuno, Ryu Yasuda, Yuichiro Kitagawa, Tetsuya Fukuta, Takahito Miyake, Haruka Okamoto, Norihide Kanda, Tomoaki Doi, Hideshi Okada, Takahiro Yoshida, Shozo Yoshida, Shinji Ogura

**Affiliations:** Advanced Critical Care Center, Gifu University Hospital, 1-1 Yanagido, Gifu, 501-1194 Japan

**Keywords:** Aortoiliac occlusive disease, Conservative treatment, Leriche syndrome, Trauma

## Abstract

**Background:**

Leriche syndrome is caused by atherosclerosis and is often characterized by symptoms such as intermittent claudication and numbness and coldness of the lower limbs. Its exact prevalence and incidence are unknown because it is a rare disease. We report a case of Leriche syndrome diagnosed incidentally on trauma pan-scan computed tomography (CT).

**Case presentation:**

A 61-year-old Asian male was driving a passenger car and had a head-on collision with a dump truck that required an emergency call. The patient was transported to our hospital in a doctor’s helicopter. Physical examination revealed the following vital signs: respiratory rate, 23 breaths per min; SpO_2_, 98% under a 10-L administration mask; pulse rate, 133 beats per min; blood pressure, 142/128 mmHg; Focused Assessment with Sonography for Trauma, positive; Glasgow Coma Scale assessment, E3V5M6; and body temperature, 35.9 °C.

Trauma pan-scan CT showed bilateral mandibular fractures, bilateral multiple rib fractures, bilateral pneumothorax, sternal fractures, hematoma around thoracic spine, small bowel perforation, mesenteric injury, right clavicle fracture, right ankle debridement injury, and thrombotic occlusion from just above the abdominal aortic bifurcation to the bilateral common iliac arteries. Although thrombotic occlusion needed to be differentiated from traumatic aortic injury, the presence of collateral blood vessels led to the diagnosis of Leriche syndrome, and conservative treatment was performed.

Damage control surgery was required for the small bowel injuries. From the second day of admission, the patient was treated with continuous intravenous heparin and prostaglandin preparations. However, impaired blood flow and reperfusion injury in the right lower extremity progressed. On the fifth day of admission, right thigh amputation was performed. The patient required renal replacement therapy for 2 weeks starting from the third day of admission.

**Conclusions:**

In this case, conservative therapy was initially chosen for Leriche syndrome. However, the complex factors in the acute phase of trauma led to development of hemorrhagic necrosis, requiring amputation of the lower extremity. Our findings indicate the need to carefully consider the unique factors affecting Leriche syndrome patients when considering treatment indications and choices for trauma.

## Background

Leriche syndrome is a progressive atherosclerotic disease affecting the abdominal aorta and iliac arteries. The risk factors for this syndrome include hypertension, hyperglycemia, hyperlipidemia, nicotine use, age, male sex, and family history. The classic clinical presentation is bilateral buttock claudication, decreased femoral pulses, and sexual dysfunction. Because vascular occlusion occurs due to disease progression, effective collateral pathways develop with time [1]. Therefore, it is a rare and chronic disease wherein patients are often asymptomatic, and its exact prevalence and incidence are unknown [2]. To the best of our knowledge, there are only a few reports of Leriche syndrome diagnosed after a trauma.

In this study, we report a case of Leriche syndrome that was diagnosed incidentally on computed tomography (CT) in a patient with multiple traumatic injuries.

## Case presentation

The patient was a 60-year-old Asian male, with no Leriche syndrome symptoms such as intermittent claudication, who was involved in a head-on collision with a dump truck while driving a passenger car. One hour after the accident, the patient was transported to our hospital in a helicopter with a physician onboard. On physical examination upon arrival, the patient had the following vital signs: respiratory rate, 23 breaths/min; SpO_2_, 98% under a 10-L/min administration mask; pulse rate, 133 breaths/min; blood pressure, 142/128 mmHg; Glasgow Coma Scale assessment, E3V5M6; and body temperature, 35.9 °C. The Focused Assessment with Sonography for Trauma result was positive in Morrison’s fossa and perisplenic region, and the patient’s hemodynamic status was approximately 1.0 on the shock index. We started a compatible blood transfusion 7 min after patient arrival. We suspected intra-abdominal hemorrhage, based on the mechanism of injury and physical examination findings. A trauma pan-scan CT performed 12 min after patient arrival demonstrated small bowel injury, mesenteric injury, and intra-abdominal hemorrhage that required damage control surgery (DCS). Laboratory blood test data on admission (shown in Table 1) identified mild peripheral circulatory failure and renal dysfunction; however, the data did not indicate any progression of anemia or coagulopathy at the time. DCS was started in the emergency department (ED) 42 min after patient arrival with a crash laparotomy. A 5-point packing with suction was performed to stop massive hemorrhage in the abdominal cavity. The mesentery had been lacerated 210 cm from the ligament of Treitz, and an incomplete tear of the small intestine was found in the same area; however, the liver and spleen showed no obvious damage. Active hemorrhage occurred from the mesenteric laceration site, which was sutured. The injured portion of the small intestine was cut using a linear cutter. No hematoma was present in the retroperitoneum. After confirming hemostasis, the operation was completed after 48 min with open abdominal management (OAM) using vacuum packing closure without gastrointestinal reconstruction. The patient was admitted to the acute critical care center.

**Table 1:** Laboratory findings on admission

<Complete blood cell counts>	
White blood cells	22,310 /μL
Red blood cells	416 × 10^4^ /μL
Hemoglobin	12.5 g/dL
Platelet	196 × 10^3^ /μL
<Coagulation Status>	
Activated partial thromboplastin time	26.2 s
Prothrombin time-international normalized ratio	0.97
Fibrinogen	215 mg/dL
Fibrin degradation product	104.6 μg/mL
D-dimer	35.1 μg/mL
<Arterial Blood Gas>	
FiO_2_	1.0
pH	7.444
PaCO_2_	30.9 mmHg
PaO_2_	154 mmHg
HCO^3−^	20.8 mmol/L
Base Excess	−1.9
Lactate	36 mg/dL
<Biochemistry>	
Total protein	5.7 g/dL
Albumin	3.5 g/dL
Aspartate transaminase	84 IU/L
Alanine transaminase	64 IU/L
Lactate dehydrogenase	341 IU/L
Alkaline phosphatase	223 IU/L
Creatinine	1.47 mg/dL
Blood urea nitrogen	24.1 mg/dL
Total bilirubin	0.4 mg/dL
Sodium	138 mEq/L
Potassium	4 mEq/L
Chloride	106 mEq/L
C-reactive protein	0.02 mg/dL
Blood sugar	198 mg/dL
Hemoglobin A1c	5.6%

**Fig. 1:**
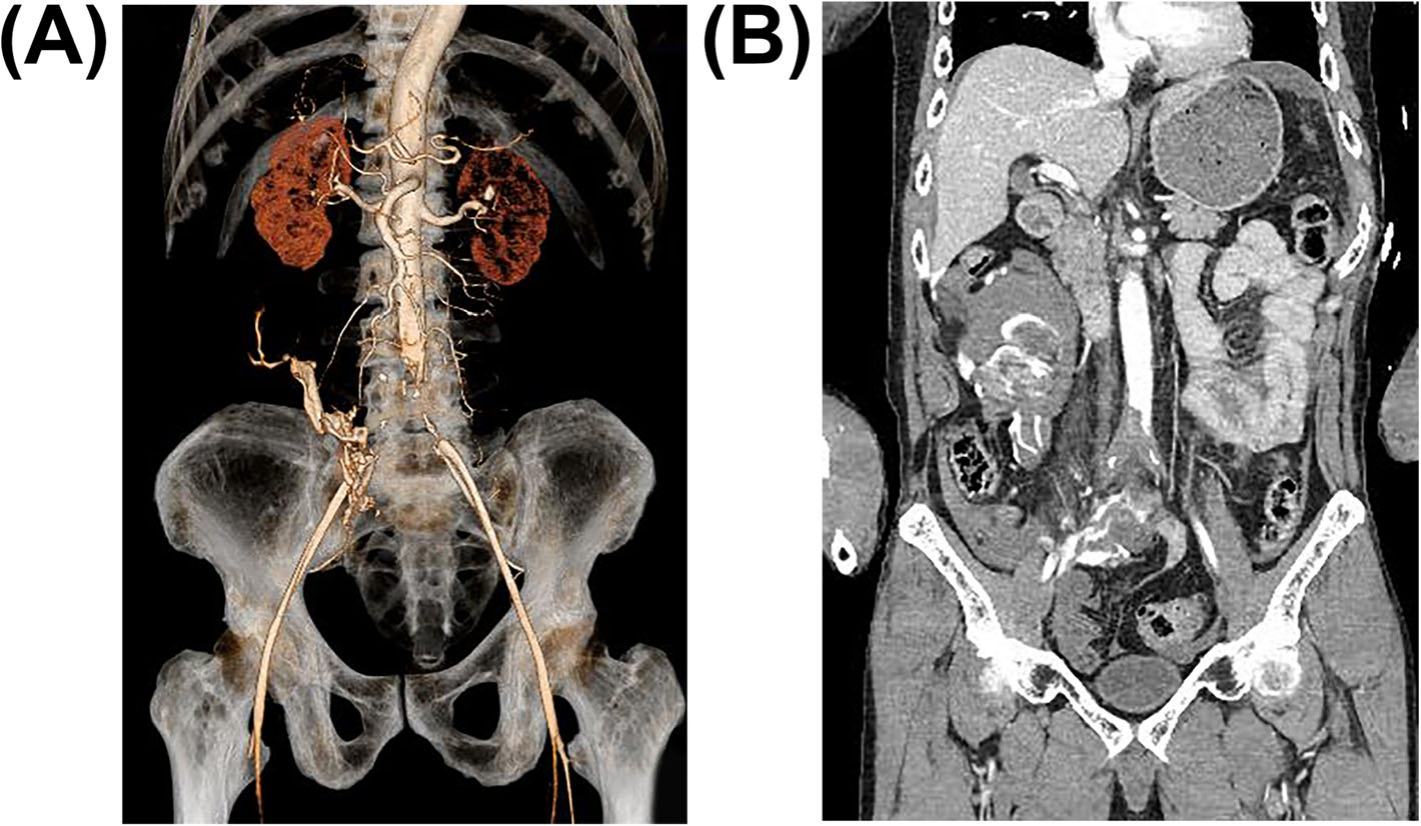
**C**oronal computed tomographic (CT) reconstructions in arterial phase images. **A** Complete occlusion just above the bifurcation of the abdominal aorta on 3D-CT image. In particular, the collateral blood pathway to the right external iliac artery can be seen. **B** Coronal contrast-enhanced CT images show disruption of blood flow due to bilateral thrombi at the aortic bifurcation and distal blood flow

Additional imaging findings were bilateral mandibular fractures, bilateral multiple rib fractures, bilateral pneumothorax, sternal fracture, hematoma around thoracic spine, right clavicle fracture, right ankle debridement injury, and thrombotic occlusion from just above the abdominal aortic bifurcation to the bilateral common iliac arteries (Fig. 1) The intraoperative findings of DCS in the ED revealed no obvious traumatic aortic injury, and a CT scan showed the presence of collateral blood vessels, leading to the diagnosis of Leriche syndrome, which is a chronic arterial occlusive disease. We tried to improve blood flow with intravenous alprostadil (prostaglandin preparation) and continuous intravenous heparin administration.

**Fig. 2:**
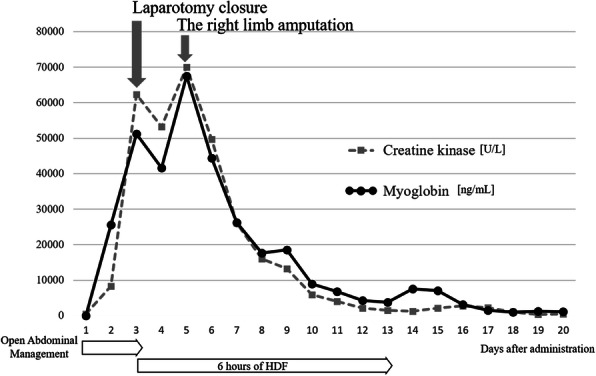
Summary of the clinical course in the reported case. **C**hange in creatine kinase (U/L) and myoglobin (ng/mL) per day after admission

Although we introduced acute renal replacement therapy to wash out creatine kinase (CK) due to the presence of acute kidney injury, grade 3 that was identified using the KDIGO criteria on day 3, the elevated CK and myoglobin levels did not improve [3]. Open-abdominal management was completed on the same day (Fig. 2). Furthermore, the findings of impaired blood flow in the right lower extremity gradually worsened. Contrast-enhanced CT of the lower extremities on day 4 showed heterogeneous hypoperfusion of the lower extremity muscles. Considering the patient’s poor general condition, the right lower limb was amputated to save the patient’s life on day 5. Thereafter, each blood test parameter began to improve, and the patient’s general condition improved. On day 14, the patient underwent tracheostomy. The patient was weaned from mechanical ventilation support on day 31, and was transferred to the general hospital ward on day 43. With rehabilitation therapy, the patient was eventually able to achieve a standing position at parallel bars under supervision and was transferred to another hospital on day 80.

## Discussion

This report highlights the findings of a patient who was diagnosed with Leriche syndrome during treatment for multiple severe traumatic injuries. We were able to save the patient’s life with multidisciplinary treatment including blood purification therapy and lower limb amputation. In this case, it was necessary to continue intermittent evaluation by frequent arterial palpation and Doppler measurements of the lower limbs during acute hemodynamic instability. As our experience following initial treatment for trauma in this case shows, chronic diseases should be correctly diagnosed and continuously evaluated, and flexible treatment strategies including early surgical intervention should be considered to improve the patient’s quality of life after lifesaving.

Leriche syndrome, also commonly referred to as aortoiliac occlusive disease (AIOD), is a subset of peripheral arterial disease (PAD) and a complication of atherosclerosis affecting the distal abdominal aorta, iliac arteries, and femoropopliteal vessels [1]. AIOD is classified as embolism and thrombosis. In general, the frequency of thrombosis (20–30%) is lower than that of embolism, and the patient may have initial intermittent claudication that is followed by well-developed collateral circulation [4]. Because 10% of patients with PAD may be asymptomatic, the exact prevalence and incidence of Leriche syndrome are unknown. The prevalence of PAD increases with age and is associated with other lifestyle-related diseases and conditions, such as hypertension, diabetes mellitus, nicotine use, hyperlipidemia, hyperglycemia, and homocysteine elevation [2, 5]. Several cases of Leriche syndrome have been discovered after surgery or isolated trauma; however, those discovered after multiple traumatic injuries are very rare. To the best of our knowledge, this is the first report of Leriche syndrome discovered in a patient with multiple traumatic injuries [6, 7].

**Fig. 3:**
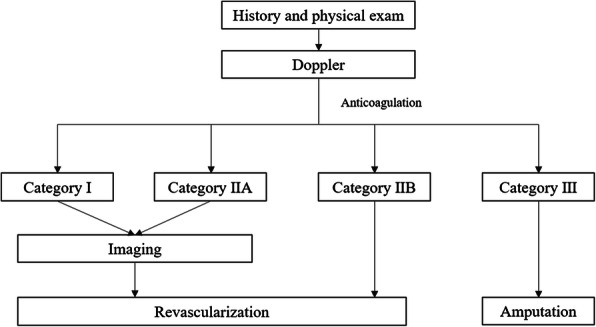
Algorithm for the management of acute lower limb arterial occlusion. **T**his algorithm was adapted from the TransAtlantic InterSociety Consensus for the management of peripheral arterial disease (TASC II) [8]

AIOD is diagnosed using CT angiography or conventional angiography. The severity classification of acute lower limb arterial occlusive disease (like AIOD) proposed by the TransAtlantic InterSociety Consensus (TASC) Working Group II is shown in Table 2 [8]. Category I patients do not require treatment. Patients in category II, especially IIb, require urgent revascularization, and for patients in category III, the lesion cannot be cured. (Fig. 3) The standard therapy is intravenous unfractionated heparin [8]. Based on the results of randomized trials, there is no clear superiority between thrombolysis and surgery for 30-day limb salvage or mortality [9]. Although the revascularization approach that has shown better long-term results is the aortobifemoral bypass, the implementation of endovascular techniques has shown superior results, thereby reducing surgical time, morbidity, and mortality in recent decades [10].

**Table 2:** Severity classification of acute lower limb arterial occlusive disease

Class	Description/prognosis	Findings		Doppler signals	
		Sensory loss	Muscle weakness	Arterial	Venous
I:Viable	Not immediately threatened	None	None	Audible	Audible
II:Threatened a:Marginal b:Immediate	Salvageable if promptly treated	Minimal (toes) or none	None	(Often) inaudible	Audible
	Salvageable with immediate revascularization	More than toes, associated with rest pain	Mild, moderate	(Usually) inaudible	Audible
III:Irreversible	Major tissue loss or permanent nerve damage inevitable	Profound, anesthetic	Profound, paralysis (rigor)	Inaudible	Inaudible

In our case, because the patient initially had no complaints of pain in the lower limbs and blood flow was visualized on Doppler, the lesion was diagnosed as category I. However, it progressed to category IIb. We suggest that a combination of factors, such as decreased collateral blood flow and coagulopathy associated with hemorrhagic shock, possible accidental collateral blood vessel injury during DCS, and increased intra-abdominal pressure associated with laparotomy closure, may have triggered the exacerbation of Leriche syndrome. We tried to improve blood flow with alprostadil (prostaglandin preparation) and continuous intravenous heparin administration. Blood flow deteriorated from the third day of hospitalization after OAM was completed; however, urgent revascularization was not possible because bypass surgery would have required revision of the abdomen, with an elevated risk of infection and bleeding, and endovascular treatment showed uncertain results in the acute phase of trauma. There were also concerns related to distal embolization. Consequently, the right lower limb was amputated on the fifth day. In hindsight, amputation may have been avoided if the OAM period had been prolonged with intensive care to stabilize the circulation and resolve the coagulopathy. The risk of suture failure increases when fascial sutures are performed after more than 5 days, and planned colostomy should be considered [6].

With aging of the population in many countries, there is a possibility that the number of Leriche syndrome cases will increase, especially in elderly patients with traumatic injuries in whom aortoiliac occlusion is discovered incidentally or when PAD is present and worsens with difficulty in communication.

We believe that it is important not only to save the lives of patients with multiple severe traumatic injuries, but also to ensure that their quality of life after treatment is as good as possible. It is important to include the evaluation of chronic disease management over time as part of the treatment strategy in the acute phase of trauma.

## Data Availability

The datasets used and/or analyzed during the current study are available from the corresponding author upon reasonable request.

## Abbreviations

AIOD: Aortoiliac occlusive disease
CK: Creatine kinase
CT: Computed tomography
DCS: Damage control surgery
ED: Emergency department
OAM: Open abdominal management
PAD: Peripheral arterial disease
TASC: TransAtlantic InterSociety Consensus
